# Effects of Ground Cover Management on Insect Predators and Pests in a Mediterranean Vineyard

**DOI:** 10.3390/insects10120421

**Published:** 2019-11-23

**Authors:** María Gloria Sáenz-Romo, Ariadna Veas-Bernal, Héctor Martínez-García, Sergio Ibáñez-Pascual, Elena Martínez-Villar, Raquel Campos-Herrera, Vicente Santiago Marco-Mancebón, Ignacio Pérez-Moreno

**Affiliations:** 1 Departament of Agriculture and Food, University of La Rioja, C/Madre de Dios 51, 26006 Logroño (La Rioja), Spain; masaenr@unirioja.es (M.G.S.-R.); ariadna.veas@unirioja.es (A.V.-B.); hector.mtz.garcia@gmail.com (H.M.-G.); elena.martinez@unirioja.es (E.M.-V.); ignacio.perez@unirioja.es (I.P.-M.); 2 Institute of Grapevine and Wine Sciences (ICVV), Finca La Grajera, 26071 Logroño (La Rioja), Spain; sibanezp@larioja.org (S.I.-P.); raquel.campos@icvv.es (R.C.-H.)

**Keywords:** abundance, cover crop, diversity, natural enemies, pests, tillage, vineyard

## Abstract

Conservative techniques, such as ground cover management, could help promote viticulture sustainability, which is a goal of conservation biological control, by providing shelter and food sources for predatory insects. A field experiment was conducted in a Mediterranean vineyard to evaluate ground cover management impacts on predatory insect and potential grapevine pest abundance and diversity, both on the ground and in the grapevine canopy. Three different ground cover management techniques (tillage, spontaneous cover and flower-driven cover) were tested for two years (2016 and 2017). Overall, the ground cover management significantly affected the abundance of important epigeal predators, of which carabids, forficulids and staphylinids were the most captured. The carabid abundances under both the cover crop treatments were found to be approximately three times higher compared with that under the tillage treatment. In contrast, the canopy insect abundance in the vineyard was similar among the treatments for both the predators and the potential grapevine pest species. These results indicate that cover crop vegetation can be used in vineyards to enhance predatory insect abundance and may improve agroecosystem resilience.

## 1. Introduction

Agriculture is an important human activity that affects ecosystem sustainability. Land use intensification impacts agroecosystem functioning by reducing biodiversity and causing shifts in functional composition [1,2,3]. Grape is a major monoculture crop worldwide with high levels of habitat disturbance due to, among other things, considerable use of agrochemicals [4]. Biological control of pests is an important ecosystem service and considered a valuable alternative to chemical control, contributing to achievement of sustainable viticulture [5,6]. 

Insects constitute a crucial component of agroecosystem biodiversity, and they are essential in maintenance of soil structure and fertility, organic matter decomposition, seed dispersion, crop pollination and pest control [4]. Predatory insects are a very important group of natural enemies of pests, and their community structure and composition have substantial impacts on biological control effectiveness [7,8]. Although native generalist predators play an important role as phytophagous population regulators in agroecosystems, their importance has not been recognised until relatively recently [9]. Nevertheless, their conservation is the core of conservation biological control (CBC) [10,11]. Some of these generalist predators, such as ground beetles, are also used as indicators of ecological sustainability because of their clear response to habitat changes, large numbers of species, ease of capture and wide distribution [12,13]. It is worth noting that both specialist and generalist predators live together in agroecosystems. For example, in vineyards, ground beetles (Coleoptera: Carabidae), earwigs (Dermaptera: Forficulidae), rove beetles (Coleoptera: Staphylinidae), ladybirds (Coleoptera: Coccinellidae), lacewings (Neuroptera: Chrysopidae), predatory bugs (Heteroptera) and other predators were found [14,15].

Insect presence is usually positively correlated with vegetation abundance and diversity [16,17]. Therefore, creating adequate vegetation infrastructure in or around crops is a sustainable measure to increase predator abundance and diversity [18,19]. Among such agroecological infrastructures, cover crops, composed of native or sown vegetation, are ideal candidates to enhance biodiversity and promote soil conservation [20]. Thus, cover crop use can affect sustainable viticulture, because it greatly influences ecosystem services and promotes CBC goals by providing favourable microclimates, shelter and food sources for predatory insects, which need pollen, floral and extrafloral nectars [21,22,23]. Several researchers have reported an increment of abundance and/or diversity of arthropod predators, as result of this kind of ground cover management in vineyards [24,25,26]. Nevertheless, increasing vegetation diversity is no guarantee of pest control, and pest species may even take advantage of benefits provided by cover crops [27]. Therefore, plant selectivity should be carefully considered to avoid promotion of pest species. 

This study is part of a research project focused on effects of different ground cover management strategies in a Mediterranean vineyard on total and functional abundance and diversity of arthropods. This study was undertaken to evaluate ground cover management impacts on predatory insect abundance and diversity in a Mediterranean vineyard. Three different ground cover management strategies were tested: (i) tillage; (ii) native vegetation; and (iii) flower mixture seeded. The overall impact on insect abundance and diversity at an order level was recently reported [14], as was their effects on predatory mites [25]. Herein, we addressed the following questions: (i) What are the predatory and potential pest insect assemblages at both the ground and grapevine canopy levels? (ii) How do ground cover management techniques impact predator and potential grapevine pest abundance and diversity? We hypothesised that cover crop use in vineyards, both composed of native vegetation or a mixture of flowering plants sown, supports abundance and diversity of natural enemies, such as insect predators, and may help meet CBC goals. In addition, we observed that cover vegetation enhanced beneficial entomofauna without promoting grapevine pests. Thus, cover crop use in vineyards may improve agroecosystem resilience.

## 2. Materials and Methods 

### 2.1. Study Site and Experimental Design

This study was performed in 2 hectares of a rain-fed vineyard in La Rioja (Northern Spain) (42°26′ N, 2°30′ W). The vineyard was planted in 1995 with the “Tempranillo” variety of *Vitis vinifera*, with a planting pattern of 2.9 m between rows and that of 1.15 m within rows. The soil texture was characterised by loam and sandy loam with low organic matter (<1%). Three soil management techniques (9 plots in total, 3 per treatment) were analysed using a completely randomised design for two years (2016 and 2017): (i) tillage; (ii) spontaneous cover; and (iii) flower-driven cover. Each plot comprised 360 vines and an area of 1200 m^2^. In the tillage treatment, the most common undervine management technique used in Spanish vineyards with bare soil (15–20 cm depth) was practised. For the spontaneous cover treatment, the ground vegetation was mowed in June once every year. The weed community was dominated by annual dicotyledonous plants with relatively short and early flowering periods and was mainly characterised by *Veronica hederifolia* (Scrophulariaceae), *Urtica dioica* (Urticaceae), *Bromus tectorum* (Poaceae), *Stellaria media* (Caryophyllaceae), *Hordeum murinum* (Poaceae), *Capsella bursa pastoris* (Brassicaceae) and *Papaver rhoeas* (Papaveraceae). Besides, the flower-driven cover treatment was sown every year in March with “Deco Vignes Anuelles” (Nova Flore, Champigné, France) (20 kg/ha). It was characterised by *Calendula officinalis* (Compositae), *Centaurea cyanus* (Asteraceae), *Cosmos bipinnatus* (Asteraceae), *Dahlia* sp. (Asteraceae), *Eschscholzia californica* (Papaveraceae), and *Lepidium* sp. (Brassicaceae). Flowers were selected that had a good balance of high-quality nectar and pollen, bright colours and gradually bloomed throughout the vegetative cycle of the vine. Furthermore, a detailed vegetation characterisation (relative abundance and diversity values) of the spontaneous and flower-driven cover treatments can be found in [25]. Finally, the vineyard management followed environmentally friendly strategies, which included mating disruption for *Lobesia botrana* Den & Schiff (Lepidoptera: Tortricidae) control and no herbicide use. Moreover, pesticides were mainly applied to control *Eotetranychus carpini* (Oudemans) (Acari: Tetranychidae), *Erysiphe necator* Schwein (“powdery mildew”) and *Plasmopara viticola* (Berk. & M.A. Curtis) Berl & De Toni (“downy mildew”) (Supplementary Table S1).

### 2.2. Insect Sampling

Insects were collected twice a month from the beginning of May to the end of September in both 2016 and 2017 (20 sampling events across both the years). The insect fauna was studied using two different sampling techniques to provide a broad understanding of the main groups of predatory and pest insects. We used pitfall traps at a ground level and vacuum sampling in the grapevine canopy. The pitfall traps consisted of a plastic bottle with a funnel, which contained 150 mL of 25% ethylene glycol, and two per plot were located along the central row under the canopy approximately 30 m apart. These were active continuously between the samplings. Additionally, the vacuum sampling was carried out with a field aspirator, InsectZooka 2888A^®^ (BioQuip Products, Rancho Dominguez, CA, USA), for 2 min per plot. All the samples were preserved in 70% ethanol at 3 °C until insect identification. Adult predatory insects were sorted to morphospecies and in specific ground beetles to genus. Potential grapevine pests were identified to species level. The insects were identified with help of Chinery, Triplehorn and Johnson, Jeannel, Herrera and Arriabita as well as Ortuño and Marcos [28,29,30,31,32].

### 2.3. Data Analyses

Relative abundance (%) (proportion of collected insects from each studied taxa of the total number) was calculated for the predatory and potential grapevine pest insects collected by the pitfall and the vacuum sampling to analyse insect community assemblages. Insect data were tested for normality (Kolmogorov–Smirnov test) and homogeneity of variances (Levene’s test), and they were log (x + 1)-transformed, when homoscedasticity was violated. To test the impact of the ground management technique, the effects of each treatment on the cumulative insect abundance and diversity were analysed by two-way ANOVA and a post-hoc Tukey’s honestly significant difference (HSD) test (α = 0.05). The dependent variables were analysed with respect to the factors: year, treatment and interaction year × treatment. The ground and canopy samples were analysed separately. A single pitfall sample was constituted by a combination of two traps per plot. All the analyses were performed in SPSS 20.0 (SPSS Statistics, SPSS Inc., Chicago, IL, USA). Biodiversity was evaluated using Hill numbers (^q^D), also known as “effective number of species” or “true diversity”, which allows for a more accurate interpretation of results [33,34]. The order of diversity (q) represents sensitivity to common and rare species. q = 0 indicates the species richness; q = 1 indicates the exponential form of the Shannon–Wiener index (H′); and q = 2 indicates the inverse of the Simpson index (λ). Additionally, figures were prepared using GraphPad Prism for Windows 8.00 (GraphPad Inc., La Jolla, CA, USA).

## 3. Results

### 3.1. Epigeal and Canopy Insect Assemblages

In total, 3560 predatory and potential pest insects were collected during the two years of study; 87.39% and 12.61% were captured using pitfall and vacuum sampling, respectively. The predators dominated the epigeal insect assemblages compared with the potential grapevine pests (99.52% vs. 0.48%). The ground beetles, the earwigs and the rove beetles were the most representative families captured by pitfall traps (67.66%, 19.67% and 3.60%, respectively). On the contrary, the ratio of the predators to the potential grapevine pests in the grapevine canopy was 6:4, mainly because of the abundance of *Empoasca vitis* (Goethe) (Hemiptera: Cicadellidae) (37.64%). Most of the predatory insect families collected by vacuum sampling belonged to Aeolothripidae (Thysanoptera), Chrysopidae, Cecidomyiidae (Diptera) and Coccinellidae (34.52%, 10.47%, 8.46% and 6.01%, respectively). Each of these natural enemy families is able to support biological control of different grapevine pests.

The richness of the predator families (*n* = 15), composed of Carabidae, Forficulidae, Aeolothripidae, Staphylinidae, Cecidomyiidae, Chrysopidae, Coccinellidae, Reduviidae (Heteroptera), Miridae (Heteroptera), Crabronidae (Hymenoptera), Vespidae (Hymenoptera), Sphecidae (Hymenoptera), Anthocoridae (Heteroptera), Geocoridae (Heteroptera) and Asilidae (Diptera), was considerably higher compared with that of the potential grapevine pest species (*n* = 4). Regarding the potential grapevine pest species, *E. vitis* was dominant (97.17%) with respect to *Altica ampelophaga* Guérin-Méneville (Coleoptera: Chrysomelidae), *Xylotrechus arvicola* (Olivier) (Coleoptera: Cerambycidae) and *Sinoxylon sexdentatum* (Olivier) (Coleoptera: Bostrichidae) (1.13%, 1.13% and 0.57%, respectively).

### 3.2. Impact of Different Ground Cover Management Techniques on Insect Abundance

**Figure 1:**
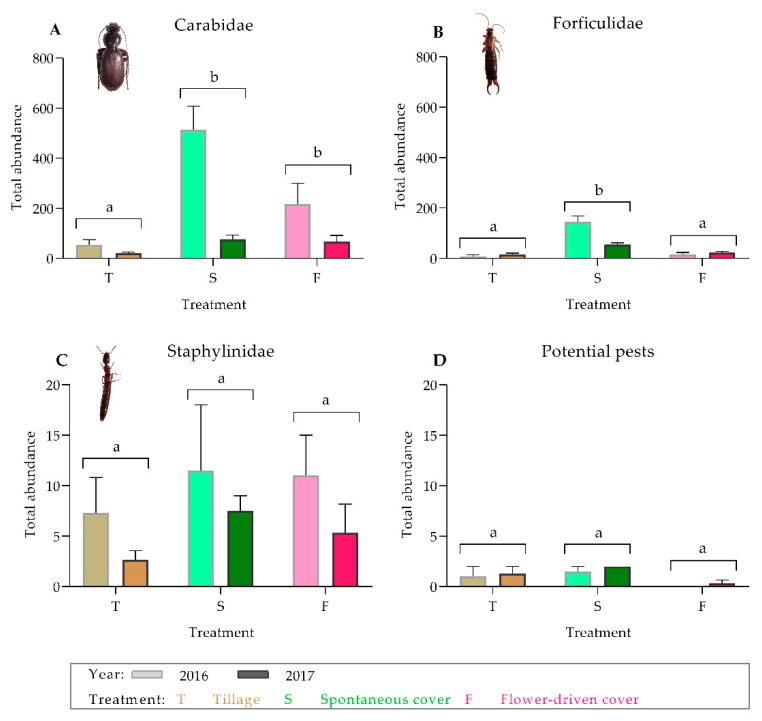
Effects of soil management on the total abundance of the predator families on the ground: (**A**) Carabidae; (**B**) Forficulidae; (**C**) Staphylinidae; and (**D**) potential grapevine pests on the ground. Values are mean (± standard error). The left bar of each couplet represents data in 2016, and the right bar represents data in 2017. Different letters indicate significant differences between the treatments, by two-way ANOVA and a Tukey’s HSD test (α = 0.05).

The ground cover management techniques significantly affected the important epigeal predator abundance, which was higher under the cover crop treatment compared with under the tillage treatment (Table 1; Figure 1). In addition, these abundances were greater under the spontaneous cover treatment than under the flower-driven cover treatment. Specifically, the cover crop treatments showed approximately three times higher abundance of carabids compared with the tillage treatment (Figure 1A), although forficulids were only significantly more abundant under the spontaneous cover treatment (Figure 1B). However, no significant differences were found among the treatments for staphylinids or the potential grapevine pests (Figure 1C,D). 

**Figure 2:**
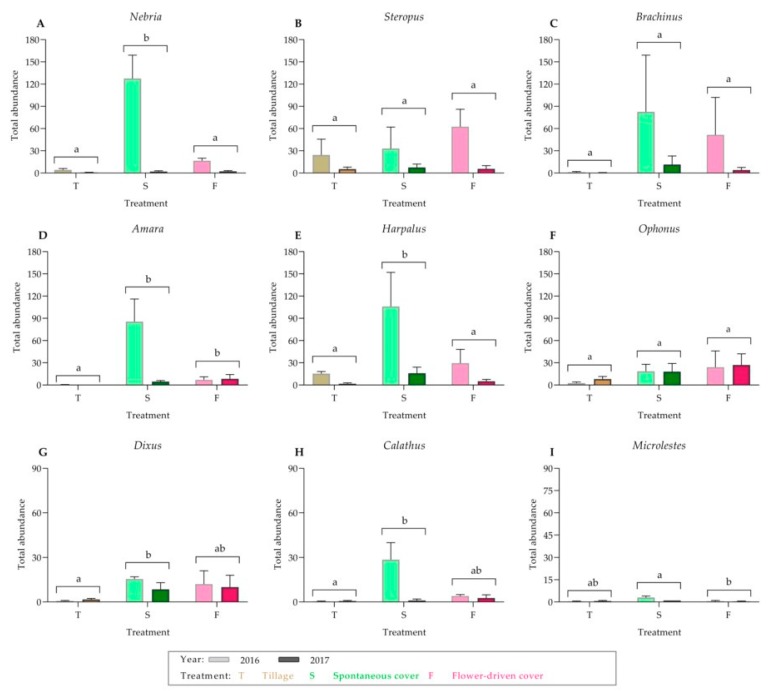
Carabidae genera captured on the ground: (**A**) *Nebria*; (**B**) *Steropus*; (**C**) *Brachinus*; (**D**) *Amara*; (**E**) *Harpalus*; (**F**) *Ophonus*; (**G**) *Dixus*; (**H**) *Calathus*; and (**I**) *Microlestes*. Values are mean (± standard error). The left bar of each couplet represents data in 2016, and the right bar represents data in 2017. Different letters indicate significant differences between the treatments, by two-way ANOVA and a Tukey’s HSD test (α = 0.05).

For the ground beetles, 20 morphospecies that belonged to nine genera on the ground were identified (Table 2; Supplementary Table S2; Figure 2). The most common carabid morphospecies was *Nebria* sp1. (15.39%), which was present along with *Steropus* sp1. (15.06%), *Brachinus* sp1. (14.68%) and *Amara* sp1. (10.55%). The other carabid morphospecies had relative abundances of less than 10%. In addition, *Harpalus* and *Ophonus* were the genera with the most morphospecies identified (*n* = 5). Moreover, *Nebria* and *Harpalus* were significantly more abundant under the spontaneous cover treatment compared with those under the other treatments. Only the *Amara* abundance was significantly greater under both the cover crop treatments. Finally, no significant differences were found among the treatments for *Steropus*, *Brachinus* and *Ophonus*. 

**Figure 3:**
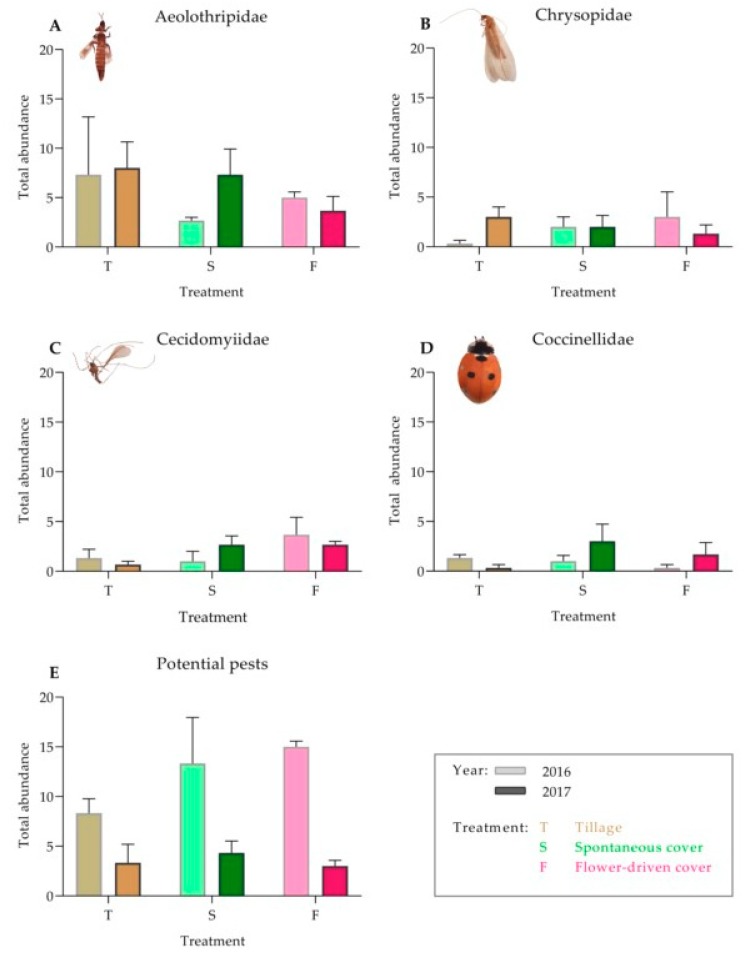
Effect of soil management on the total abundance of the predator families in the canopy: (**A**) Aeolothripidae; (**B**) Chrysopidae; (**C**) Cecidomyiidae; (**D**) Coccinellidae; and (**E**) potential grapevine pests in the grapevine canopy. Values are mean (± standard error). The left bar of each couplet represents data in 2016, and the right bar represents data in 2017.

In contrast to the epigeal fauna, the canopy insect abundance in the vineyard was similar among the treatments for both the predators and the potential grapevine pest species (Table 3; Figure 3). For the predatory insects, higher abundances of Chrysopidae, Cecidomyiidae and Coccinellidae were observed under both the cover crop treatments compared with those under the tillage treatment, although these differences were not statistically significant. 

**Figure 4:**
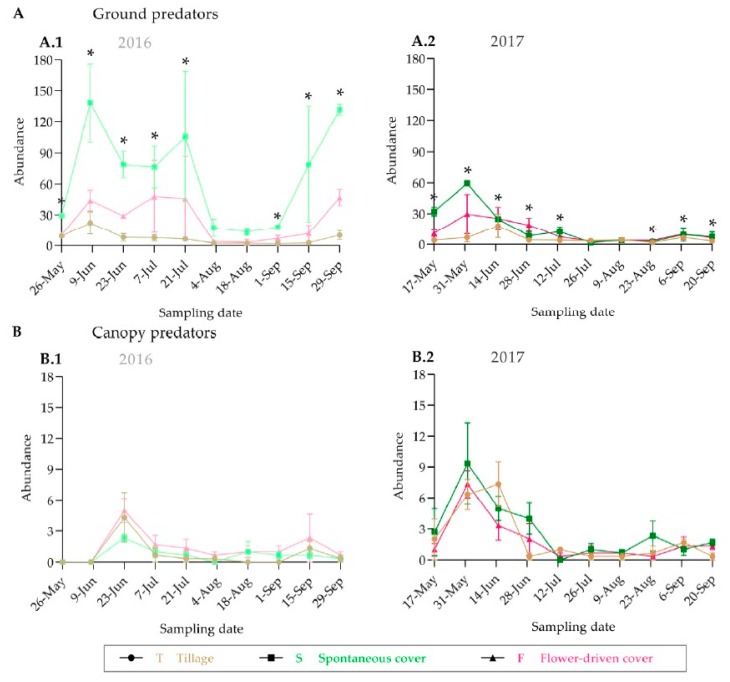
Population dynamics of predators on the ground (**A**) and in the grapevine canopy (**B**). An asterisk indicates significant differences between treatments, by ANOVA and Tukey-HSD test (α = 0.05).

**Figure 5:**
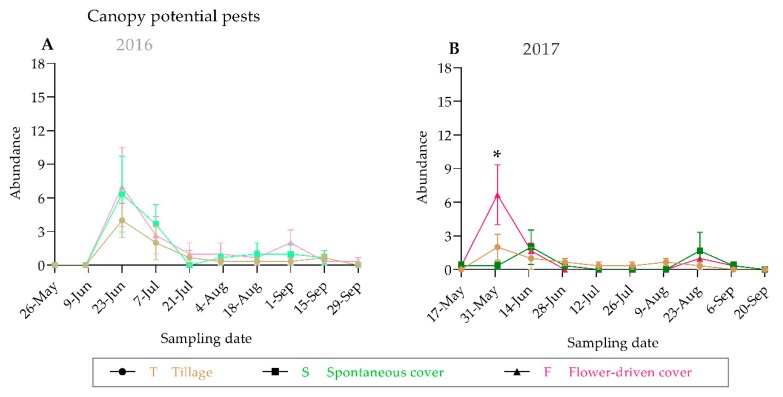
Population dynamics of potential grapevine pests in the grapevine canopy in 2016 (**A**) and 2017 (**B**). An asterisk indicates significant differences between treatments, by ANOVA and Tukey-HSD test (α = 0.05).

We observed similar trends in the population dynamics of the predators and the potential grapevine pests between the years both on the ground and in the grapevine canopy, with a higher abundance at the beginning of the grapevine vegetative cycle (Figure 4 and Figure 5; Supplementary Table S3). The greatest epigeal predator abundance was observed at the beginning of June, which coincides with the grape phenological stage 17-H (separate floral buttons). The abundance of the epigeal predators collected in 2016 at the same phenological stage was nearly two times higher than that in 2017. In fact, half of the sampling dates significantly differed among years (Supplementary Table S3). For almost all the samplings, the spontaneous cover treatment showed the highest epigeal predator abundance values, which were statistically significant except in August (Figure 4; Supplementary Table S3). In contrast, the grapevine canopy predator and potential grapevine pest abundances were quite similar between the years, and no significant differences were recorded among the treatments, except for one sampling date (31 May 2017), in the case of the potential grapevine pests (Figure 4 and Figure 5; Supplementary Table S3). The potential grapevine pests and the predaceous insects showed overlap in habitat use during the fruit-growing season. 

### 3.3. Effects of Different Ground Cover Management Techniques on Insect Diversity

The observed predatory insect diversity was higher on the ground than in the grapevine canopy; however, the opposite was true for the potential pests (Table 4; Supplementary Table S4). Of the families studied, Carabidae showed the highest diversity values, both on the ground and in the grapevine canopy. The highest carabid richness was recorded under the spontaneous cover treatment. The effective number of the ground beetles calculated for three diversity levels (^0^D, ^1^D and ^2^D) increased as the order of diversity (q) decreased, which denoted a high degree of dominance in the community; decreases, as q increased, were stronger under the cover crop treatments, where the ratio between ^2^D and ^0^D was around 3. However, this trend was not observed for the grapevine canopy predators, where the number of the common species (^2^D) was quite similar to the richness (^0^D). Additionally, the flower-driven cover treatment showed greater, although not significantly different, diversity of cecidomyiids than the other treatments. Moreover, there was higher diversity of potential pests in the grapevine canopy, although we only found differences at the ground level. 

## 4. Discussion

The grapevines occur at the centre of complex communities, with a wide range of insects at both the ground and canopy levels. We expected that the ground cover management influences the insect predatory population both on the ground and in the grapevine canopy, but we only observed a significant effect on some epigeal predator taxa. Nevertheless, we cannot compare results between the ground and canopy levels because of using two different techniques for sampling. 

### 4.1. Epigeal Predators

The vineyard, where this study was conducted, supported a diverse predatory insect assemblage. The ground beetles were the most abundant and diverse insects captured on the ground, which is consistent with the findings reported by Kromp et al. [12,35] in other crops. Earwigs were the second most abundant but only represented by one species, *Forficula auricularia* Linnaeus, 1758 (Dermaptera: Forficulidae), which is also known as the European earwig and is an important omnivorous predator in vineyards. The spontaneous cover vegetation had a significant impact on the abundance of both the families compared with the tillage treatment, which is consistent with the findings of Danne et al., Irvin et al. and Sharley et al. [36,37,38]; this could be explained by tilling effects, such as habitat disturbance, litter layer removal, microclimate condition alterations as well as shelter and food availability reduction [39], which have strong impacts on insects that live on the soil surface. However, no differences among the treatments were observed for the rove beetles, one of the most ecologically important predaceous insects in agroecosystems; this is consistent with the observations of Bohac [40], who reported that agricultural measures, such as tillage, have a lower influence on staphylinids compared with others factors (e.g., landscape factors).

Carabids are considered an ecologically important family of natural enemies of pests [12] and key contributors to biocontrol in agroecosystems [41] because of their broad diet, which allows them to persist and prevent pest outbreaks despite seasonal disturbance [42]. Most carabids are polyphagous predators, and both larval and adult forms are able to feed on pests such as lepidopteran larvae, aphids and slugs [43]. In addition, some species can also feed on leaves, seeds, fruits and fungi [43]. 

The ground beetle communities in the studied vineyard were dominated (>85%) by six genera: *Harpalus*, *Nebria*, *Steropus*, *Brachinus*, *Ophonus* and *Amara*. Three genera (*Harpalus*, *Ophonus* and *Amara*) belong to the tribe Harpalini and are well known as true granivores [44]. Several species of *Harpalus* are known to be involved in seed regulation in vineyards without any seed preferences [45]. However, specific affinities have been reported for *Ophonus* and *Amara*, such as Apiaceae and Poaeceae, respectively [46,47]. Both the plant families were recorded in the spontaneous cover treatment (0.10% and 14.80%, respectively) [14]. This higher relative abundance of Poaceae might be positively correlated with a higher abundance of *Amara* under the spontaneous cover treatment. In addition, most of the Carabidae genera were more abundant under both the cover crop treatments than under the tillage treatment, even if differences were only significant in the case of *Amara*. *Amara* may have been more abundant, because it is a spermophagous genus [48], and seeds retained on the surface of the cover crop treatments may provide an important source of food. However, several carabid genera were only significantly more abundant under the spontaneous cover treatment, such as *Nebria*, *Harpalus*, *Dixus* and *Calathus*; this finding is consistent with those of other studies [49,50], which reported that ground beetles do not directly feed on floral resources and that native vegetation may increase food availability for them.

There are various factors, such as ground beetle body size, mobility and trophic levels, which are often considered to be potentially essential in carabid responses to habitat quality [51,52]. In the studied vineyard, the most abundant genera were *Harpalus* and *Nebria*, and both were significantly more abundant under the spontaneous cover treatment. *Harpalus* may be abundant, because weeds and variety of seeds of grasses provide a great amount of their food sources. Alternatively, *Nebria* may be abundant as a result of native cover vegetation effects on microclimatic conditions (temperature and humidity) and shelter, because they have hygrophilic and photophobic tendencies [32]. Alternatively, some authors suggested that larger carabids (size ≥ 15 mm) are negatively associated with disturbed habitats [52,53,54,55]. However, no differences were found among the treatments relative to the abundance of *Steropus* (large carabids) in the studied vineyard, potentially because this genus is able to tolerate a wide range of environments [56]. Conversely, these differences were observed in the abundance of *Microlestes* (small carabids), which were more abundant under the treatment with less disturbance. Nevertheless, *Microlestes* abundance did not differ under the tillage treatment compared with under the cover crop treatments, potentially because they are able to tolerate sunlight and sudden changes in humidity and temperature [32]. 

### 4.2. Grapevine Canopy Predators

The studied cover crop treatments did not significantly affect the grapevine canopy predaceous insect abundance. Cover vegetation in vineyards can provide shelter, nectar, alternative prey and pollen, which support insect populations [57]. Several authors have reported that floral nectar and pollen also are highly attractive to lacewings and coccinellids [58], but differences among the treatments were not found in this study. *Chrysoperla carnea* (Stephens, 1836) (Neuroptera: Chrysopidae) was the main lacewing captured; it is a polyphagous predator in its larval form but only feeds on sugary substances and pollen in its adult form. Similarly, the larvae of several cecidomyiid species are predators, especially of aphids, and can also attack mealybugs, mites and other small arthropods, whereas adults feed on floral sources. The Cecidomyiid abundances were around two and three times higher under the spontaneous cover and flower-driven cover treatments, respectively, compared with that under the tillage treatment, although not significantly. Among Coleoptera, the most abundant predatory insect family was Coccinellidae, which was mostly represented by *Coccinella (Coccinella) septempunctata* Linnaeus, 1758, *Scymnus (Scymnus) interruptus* (Goeze, 1777), *Adonia variegata* (Goeze, 1777), *Coccidula rufa* (Herbst, 1783) and *Propylea quatuordecimpunctata* (Linnaeus, 1758). Most of these insects attack aphids, although they can also feed on the eggs of lepidopterans, such as *L. botrana*. Ladybirds may be more abundant under both the spontaneous cover and flower-driven cover treatments compare with that under the tillage treatment, because *C. septempunctata* lives in the herbaceous layer, which is less than half a meter in length, and some authors [59] have reported that *Centaurea cyanus* Linnaeus, 1753 (Asteraceae) is positively correlated with their presence. The Coccinellid abundance was nearly two times higher under the spontaneous cover treatment than under the tillage treatment, but not significantly. Besides, we did not observe differences between the spontaneous cover and flower-driven cover treatments in relation with the ladybirds abundance. These results are in line with the published paper by Burgio et al. [24] in vineyard but in contrast with other authors [58,60] that reported a positive effect of flowering plants on Coccinellidae.

### 4.3. Pest Assemblages

The presence of the potential grapevine pests was negligible at the ground level, but they did occur in the grapevine canopy. The main pest in Mediterranean and European vineyards, L. *botrana*, was not captured in this study. This indicates that mating disruption, in addition to being an environmentally friendly technique, is efficient to control this pest. However, we recorded the dominance of *E. vitis*, which is a polyphagous cicadellid. It is considered a secondary pest, which can be found on both grapevines and weeds [61]. Species of coccinellids, neuropterans (e.g., *C. carnea*) and heteropterans (e.g., *Orius* spp. (Hemiptera: Anthocoridae)) have been cited as predators of *E. vitis*. Otherwise, only three coleopterans occasionally were captured in vineyards and are considered secondary pests (*A. ampelophaga, X. arvicola*, and *S. sexdentatum*). No significant differences among the treatments were found relative to the potential pest abundance. Therefore, although diverse cover vegetation can support many phytophagous insects, according to the data reported by Sáenz-Romo et al. and Siemann et al. [14,62], it does not seem to enhance potential grapevine pest species.

### 4.4. Insect Population Dynamics

With respect to the population dynamics, the epigeal predator abundance showed strong annual variability, possibly due to abiotic factors (mainly temperature and relative humidity), which were harsher in 2017 than in 2016 [25]. Nevertheless, almost no differences were found at the grapevine canopy level; this may be explained by microclimatic conditions, which are more favourable because of the grapevine leaves effect. 

Alternatively, although the total epigeal predator abundance was significantly higher under the spontaneous cover treatment on almost all the sampling dates, it was observed that the grass mowing, carried out in the beginning of June, caused the population decline. This finding is consistent with those of Rouabah et al., Thorbek and Bilde as well as Woodcock et al. [63,64,65], who reported that reduction in vegetation height has a clear impact on abundance of carabid and staphylinid beetles. Furthermore, reduction of epigeal predators in mid-summer may be caused by temporarily depressed ground beetle activity densities due to high nightly temperatures [43]. Moreover, according to Sáenz-Romo et al. and Rebek et al. [14,66], spontaneous vegetation biomass can attract predaceous insects and alternative prey in vineyards, even when flowers are not in bloom. Thus, spontaneous vegetation cover in vineyards might be associated with providing benefits to predaceous insects throughout the growing period, which is consistent with the findings reported by Thomson and Hoffmann [67]. 

### 4.5. Diversity Values

Most agroecosystem biodiversity resides in the soil [68], and this is particularly true for insects. Even though intensification of agricultural practices such as tilling has been reported to be important drivers of biodiversity loss in agroecosystems [69,70], no significant differences were found among the treatments in most of the predatory families studied. Nevertheless, it was observed that the spontaneous cover treatment increased the carabid richness (^0^D). This result confirms the possibility that carabid morphospecies richness is positively correlated with higher vegetation diversity, which was also reported by other researchers [45,52,71,72]. Thereby, a cover crop canopy seems to be a key factor that influences both abundance and diversity of epigeal predators such as ground beetles. According to Melnychuck et al. [73], epigeal predator diversity tends to be higher under an herbaceous cover of grasses, because spring growth provides early coverage, as was observed in the studied vineyard. 

## 5. Conclusions

Overall, the insect communities were influenced by the ground cover management techniques in the studied vineyard. It impacted the insect predators on the ground but not in the grapevine canopy. The cover crop vegetation enhanced beneficial entomofauna, especially carabids and forficulids, without promoting potential grapevine pest species. In particular, the spontaneous cover vegetation increased both the abundance and the diversity of ground beetles. More specifically, it significantly impacted the abundance of the carnivorous genus *Nebria* in comparison with the tillage and flower-driven treatments. Thus, in fact, establishment of long-term vegetation cover could improve agroecosystem resilience, and management of spontaneous cover vegetation seems to be the most interesting strategy for implementing CBC in vineyards.

## Figures and Tables

**Table 1:** Two-way ANOVA results of the total abundance of the main predator families and the total potential grapevine pests on the ground. Significant differences are highlighted in bold.

Taxa	Year	Treatment	Year × Treatment
Carabidae	F_1,16_ = 25.33, ***p* = 0.001**	F_2,16_ = 17.06; ***p* = 0.001**	F_2,16_ = 1.35; *p* = 0.31
Forficulidae	F_1,16_ = 0.14; *p* = 0.72	F_2,16_ = 11.79; ***p* = 0.003**	F_2,16_ = 2.03; *p* = 0.19
Staphylinidae	F_1,16_ = 2.99; *p* = 0.12	F_2,16_ = 1.00; *p* = 0.41	F_2,16_ = 0.03; *p* = 0.97
Potential pests	F_1,16_ = 0.72; *p* = 0.42	F_2,16_ = 3.11; *p* = 0.09	F_2,16_ = 0.01; *p* = 0.99

**Table 2:** Two-way ANOVA results of the total abundance of the Carabidae genera captured on the ground. Significant differences are highlighted in bold.

Genus	Year	Treatment	Year × Treatment
*Nebria*	F_1,16_ = 68.39, ***p* < 0.001**	F_2,16_ = 18.31; ***p* = 0.001**	F_2,16_ = 9.19; ***p* = 0.007**
*Steropus*	F_1,16_ = 3.18; *p* = 0.11	F_2,16_ = 0.59; *p* = 0.57	F_2,16_ = 0.74; *p* = 0.51
*Brachinus*	F_1,16_ = 2.16; *p* = 0.18	F_2,16_ = 2.67; *p* = 0.12	F_2,16_ = 0.40; *p* = 0.68
*Amara*	F_1,16_ = 8.38; ***p* = 0.02**	F_2,16_ = 23.22; ***p* = 0.001**	F_2,16_ = 5.06; ***p* = 0.03**
*Harpalus*	F_1,16_ = 22.13; ***p* = 0.001**	F_2,16_ = 7.83; ***p* = 0.01**	F_2,16_ = 0.09; *p* = 0.92
*Ophonus*	F_1,16_ = 0.67; *p* = 0.43	F_2,16_ = 2.21; *p* = 0.17	F_2,16_ = 0.31; *p* = 0.74
*Dixus*	F_1,16_ = 0.24; *p* = 0.64	F_2,16_ = 6.77; ***p* = 0.02**	F_2,16_ = 0.70; *p* = 0.52
*Calathus*	F_1,16_ = 9.35; ***p* = 0.01**	F_2,16_ = 6.77; ***p* = 0.02**	F_2,16_ = 6.23; ***p* = 0.02**
*Microlestes*	F_1,16_ = 0.80; *p* = 0.39	F_2,16_ = 4.90; ***p* = 0.04**	F_2,16_ = 1.61; *p* = 0.25

**Table 3:** Two-way ANOVA results of the total abundance of the main predator families and the total potential grapevine pests in the canopy. Significant differences are highlighted in bold.

Taxa	Year	Treatment	Year × Treatment
Aeolothripidae	F_1,18_ = 0.32, *p* = 0.59	F_2,18_ = 0.74; *p* = 0.50	F_2,18_ = 0.56; *p* = 0.59
Chrysopidae	F_1,18_ = 0.95, *p* = 0.76	F_2,18_ = 0.07; *p* = 0.93	F_2,18_ = 1.36; *p* = 0.29
Cecidomyiidae	F_1,18_ = 0.01, *p* = 0.99	F_2,18_ = 2.43; *p* = 0.13	F_2,18_ = 1.08; *p* = 0.37
Coccinellidae	F_1,18_ = 1.06, *p* = 0.32	F_2,18_ = 0.94; *p* = 0.42	F_2,18_ = 1.46; *p* = 0.27
Potential pests	F_1,18_ = 23.22, ***p* < 0.001**	F_2,18_ = 1.31; *p* = 0.31	F_2,18_ = 1.27; *p* = 0.32

**Table 4:** Hill numbers of the predatory and the potential pest insects, both on the ground and at the canopy level. Data are shown as mean (± standard error).

Observed Diversity (^q^D)		Tillage	Spontaneous Cover	Flower-Driven Cover
**Ground** **level**				
Carabidae	^0^D	11.5 (1.33) ^a^	20.25 (1.44) ^b^	16.20 (3.06) _ab_
	^1^D	6.89 (1.30) ^a^	10.16 (1.30) ^a^	7.32 (1.42) ^a^
	^2^D	4.88 (1.14) ^a^	6.95 (1.29) ^a^	4.86 (1.10) ^a^
Forficulidae	^0^D	1.00 (0.00) ^a^	1.00 (0.00) ^a^	1.00 (0.00) ^a^
	^1^D	1.00 (0.00) ^a^	1.00 (0.00) ^a^	1.00 (0.00) ^a^
	^2^D	1.00 (0.00) ^a^	1.00 (0.00) ^a^	1.00 (0.00) ^a^
Staphylinidae	^0^D	1.83 (0.54) ^a^	3.17 (0.79) ^a^	3.00 (0.73) ^a^
	^1^D	1.79 (0.54) ^a^	2.70 (0.60) ^a^	2.71 (0.63) ^a^
	^2^D	1.76 (0.54) ^a^	2.40 (0.50) ^a^	2.50 (0.60) ^a^
Potential pests	^0^D	0.17 (0.17) ^ab^	0.83 (0.31) ^b^	0.00 (0.00) ^a^
	^1^D	0.17 (0.17) ^ab^	0.83 (0.31) ^b^	0.00 (0.00) ^a^
	^2^D	0.17 (0.17) ^ab^	0.83 (0.31) ^b^	0.00 (0.00) ^a^
**Canopy level**				
Aeolothripidae	^0^D	2.33 (0.49) ^a^	2.00 (0.36) ^a^	2.16 (0.31) ^a^
	^1^D	2.11 (0.41) ^a^	1.88 (0.33) ^a^	1.97 (0.32) ^a^
	^2^D	1.98 (0.36) ^a^	1.80 (0.30) ^a^	1.85 (0.32) ^a^
Chrysopidae	^0^D	0.67 (0.21) ^a^	0.83 (0.17) ^a^	0.67 (0.21) ^a^
	^1^D	0.67 (0.21) ^a^	0.83 (0.17) ^a^	0.67 (0.21) ^a^
	^2^D	0.67 (0.21) ^a^	0.83 (0.17) ^a^	0.67 (0.21) ^a^
Cecidomyiidae	^0^D	0.83 (.031) ^a^	1.33 (0.49) ^a^	2.17 (0.31) ^a^
	^1^D	0.81 (0.29) ^a^	1.27 (0.48) ^a^	2.11 (0.31) ^a^
	^2^D	0.80 (0.28) ^a^	1.23 (0.47) ^a^	2.08 (0,32) ^a^
Coccinellidae	^0^D	0.67 (0.21) ^a^	0.83 (0.17) ^a^	0.67 (0.21) ^a^
	^1^D	0.67 (0.21) ^a^	0.83 (0.17) ^a^	0.67 (0.21) ^a^
	^2^D	0.67 (0.21) ^a^	0.83 (0.17) ^a^	0.60 (0.28) ^a^
Potential pests	^0^D	1.17 (0.17) ^a^	1.17 (0.17) ^a^	1.00 (0.00) ^a^
	^1^D	1.06 (0.06) ^a^	1.17 (0.17) ^a^	1.00 (0.00) ^a^
	^2^D	1.03 (0.03) ^a^	1.17 (0.17) ^a^	1.00 (0.00) ^a^

Different letters indicate significant differences between the treatments, by two-way ANOVA and a Tukey’s HSD test (α = 0.05).

## Supplementary Materials

The following are available online at https://www.mdpi.com/2075-4450/10/12/421/s1, Table S1: Pesticide treatments applied to the pest and disease control, Table S2: Size, relative abundance and statistical results (two-way ANOVA) of Carabidae morphospecies abundance. Significant differences are highlighted in bold, Table S3: Two-way ANOVA results of the population dynamics of the predators both on the ground and in the canopy and the potential pests in the grapevine canopy. Significant differences are highlighted in bold, Table S4: Two-way ANOVA results of predator biodiversity values. Significant differences are highlighted in bold.
