# Supplementary material for: Effects of Ground Cover Management on Insect Predators and Pests in a Mediterranean Vineyard
Source: Insects. 2019 Nov 23;10(12):421. doi: 10.3390/insects10120421 (PMC6956331; doi:10.3390/insects10120421)
Supplement: Supplementary file 1 [file insects-10-00421-s001.zip › Table S1.docx]

**Table S1.** Pesticide treatments applied to pest and disease control.

| **Active ingredients** | **Dose/ha** | **Date** |
| --- | --- | --- |
| Sulfur powder | 10 kg/ha | 28 May 2016 |
| Myclobutanil 12.5% p/v + (Folpet 40% + Metalaxil 10%) | 300 cc/ha + 1.5 l/ha | 3 June 2016 |
| (Folpet 37.5% + Iprovalicarb 6%) + (Fluopyram 20% + Tebuconazol 20%) | 2 kg/ha + 350 cc/ha | 24 June 2016 |
| Sulfur powder | 25 kg/ha | 30 June 2016 |
| (Dimetomorf 12% + Piraclostrobin 6.7%) + Spirodiclofen 24% | 1.25 kg/ha + 200 cc/ha | 16 July 2016 |
| Quinoxifen 25% p/v + (Cimoxanilo 3% + Copper 22.5%) | 300 cc/ha + 3.5 l/ha | 2 August 2016 |
| (Folpet 37.5% + Iprovalicarb 6%) + (Fluopyram 20% + Tebuconazol 20%) | 1.5 kg/ha + 300 cc/ha | 26 May 2017 |
| Sulfur powder | 20 kg/ha | 8 June 2017 |
| Dimetomorf 12% + Piraclostrobin 6.7% | 1.25 kg/ha | 20 June 2017 |
| Sulfur powder | 25 kg/ha | 30 June 2017 |
| (Cimoxanilo 3% + Copper 15% + Mancozeb 10% WP) + Ciflufenamid 3% + Difenoconazol 6% p/v + Abamectin 1.8% p/v | 3 kg/ha + 0.6 l/ha + 1 l/ha + 0.6 l/ha | 13 July 2017 |
| Cimoxanilo 3% + Copper 15% + Mancozeb 10% WP + Quinoxifen 25% p/v. | 3 kg/ha + 250 cc/ha | 2 August 2017 |
