# Supplementary material for: Effects of Ground Cover Management on Insect Predators and Pests in a Mediterranean Vineyard
Source: Insects. 2019 Nov 23;10(12):421. doi: 10.3390/insects10120421 (PMC6956331; doi:10.3390/insects10120421)
Supplement: Supplementary file 1 [file insects-10-00421-s001.zip › Table S2.docx]

**Table S2.** Size, relative abundance and statistical results (two-way ANOVA) of Carabidae morphospecies abundance. Significant differences are highlighted in bold.

| **Morphospecies** | **Size (mm)** | **RA (%)** | **Year** | **Treatment** | **Treatment (T, S, F)^a^** | **Year x Treatment** |
| --- | --- | --- | --- | --- | --- | --- |
| *Nebria* sp1. | 15 | 15.39 | F_1_,_16_= 68.39, **P < 0.001** | F_2_,_16_ = 18.31, **P = 0.001** | a, b, a | F_2,16_ = 9.19, **P = 0.007** |
| *Steropus* sp1. | 20 | 15.06 | F_1_,_16_ = 3.18, P = 0.11 | F_2_,_16_ = 0.59, P = 0.57 | a, a, a | F_2,16_ = 0.74, P = 0.51 |
| *Brachinus* sp1. | 9 | 14.68 | F_1_,_16_ = 2.16, P = 0.18 | F_2_,_16_ = 2.67, P = 0.12 | a, a, a | F_2,16_ = 0.40, P = 0.68 |
| *Amara* sp1. | 10 | 10.55 | F_1_,_16_ = 8.38, **P = 0.02** | F_2_,_16_ = 23.22, **P = 0.001** | a, b, b | F_2,16_ = 5.06, **P = 0.03** |
| *Harpalus* sp3. | 10 | 7.60 | F_1_,_16_ = 44.34; **P < 0.001** | F_2_,_16_ = 19.40, **P = 0.001** | a, b, a | F_2,16_ = 0.19, P = 0.83 |
| *Harpalus* sp2. | 12 | 7.20 | F_1_,_16_ = 14.30; **P = 0.004** | F_2_,_16_ = 6.03, **P = 0.02** | a, b, a | F_2,16_ = 0.21, P = 0.81 |
| *Ophonus* sp2. | 10 | 5.51 | F_1_,_16_ = 0.01, P = 0.99 | F_2_,_16_ = 0.84, P = 0.46 | a, a, a | F_2,16_ = 0.02, P = 0.98 |
| *Ophonus* sp1. | 9 | 4.04 | F_1_,_16_ = 2.63, P = 0.14 | F_2_,_16_ = 0.63, P = 0.55 | a, a, a | F_2,16_ = 1.32, P = 0.32 |
| *Dixus* sp1. | 15 | 2.99 | F_1_,_16_ = 0.68, P = 0.43 | F_2_,_16_ = 6.71, **P = 0.02** | a, b, ab | F_2,16_ = 0.22, P = 0.81 |
| *Calathus* sp2. | 12 | 2.95 | F_1_,_16_ = 7.86, **P = 0.02** | F_2_,_16_ = 6.14, **P = 0.02** | a, b, ab | F_2,16_ = 5.64, **P = 0.03** |
| *Dixus* sp2. | 13 | 2.23 | F_1_,_16_ = 0.20, P = 0.67 | F_2_,_16_ = 4.35, P = 0.05 | a, a, a | F_2,16_ = 0.97, P = 0.42 |
| *Harpalus* sp4. | 9 | 1.76 | F_1_,_16_ = 24.04, **P = 0.001** | F_2_,_16_ = 8.80, **P = 0.008** | a, b, a | F_2,16_ = 3.06, P = 0.10 |
| *Ophonus* sp3. | 10 | 1.38 | F_1_,_16_ = 0.01, P = 0.97 | F_2_,_16_ = 2.56, P = 0.13 | a, a, a | F_2,16_ = 0.48, P = 0.63 |
| *Calathus* sp1. | 12 | 1.28 | F_1_,_16_ = 12.25, **P = 0.007** | F_2_,_16_ = 7.85, **P = 0.01** | a, b, a | F_2,16_ = 6.44, **P = 0.02** |
| *Harpalus* sp1. | 10 | 1.28 | F_1_,_16_ = 14.61, **P = 0.004** | F_2_,_16_ = 0.33, P = 0.72 | a, a, a | F_2,16_ = 0.61, P = 0.56 |
| *Harpalus* sp5. | 10 | 0.48 | F_1_,_16_ = 3.36, P = 0.10 | F_2_,_16_ = 0.26, P = 0.78 | a, a, a | F_2,16_ = 0.26, P = 0.78 |
| *Microlestes* sp1. | 4 | 0.43 | F_1_,_16_ = 19.44, **P = 0.002** | F_2_,_16_ = 5.51, **P = 0.03** | a, b, a | F_2,16_ = 5.51, **P = 0.03** |
| *Ophonus* sp5. | 8 | 0.33 | F_1_,_16_ = 1.29, P = 0.29 | F_2_,_16_ = 0.46, P = 0.64 | a, a, a | F_2,16_ = 0.46, P = 0.64 |
| *Ophonus* sp4. | 4 | 0.29 | F_1_,_16_ = 11.47, **P = 0.01** | F_2_,_16_ =3.40, P = 0.08 | a, a, a | F_2,16_ = 3.40, P = 0.08 |
| *Microlestes* sp2. | 3 | 0.28 | F_1_,_16_ = 10.80, **P = 0.01** | F_2_,_16_ = 0.82, P = 0.47 | a, a, a | F_2,16_ = 0.82, P = 0.47 |

**^a^**Treatment (T, S, F): T = Tillage; S = Spontaneous cover; F = Flower-driven cover

Different letter indicates significant differences between treatments, by Two-way ANOVA and Tukey-HSD test (α = 0.05).
