# Supplementary material for: Effects of Ground Cover Management on Insect Predators and Pests in a Mediterranean Vineyard
Source: Insects. 2019 Nov 23;10(12):421. doi: 10.3390/insects10120421 (PMC6956331; doi:10.3390/insects10120421)
Supplement: Supplementary file 1 [file insects-10-00421-s001.zip › Table S3.docx]

**Table S3.** Two-way ANOVA results of the population dynamics of the predators both on the ground and in the canopy and the potential pests in the grapevine canopy. Significant differences are highlighted in bold.

| **Sampling dates** | **Year** | **Treatment** | **Treatment (T, S, F) ^a^** | **Year x Treatment** |
| --- | --- | --- | --- | --- |
| **A. Ground predators** |  |  |  |  |
| 1 | F_1,16_ = 1.43; P = 0.26 | F_2,16_ = 18.76; **P = 0.001** | a, b, a | F_2,16_ = 1.61; P = 0.25 |
| 2 | F_1,16_ = 4.19; P = 0.07 | F_2,16_ = 7.40; **P = 0.01** | a, b, ab | F_2,16_ = 0.22; P = 0.81 |
| 3 | F_1,16_ = 0.53; P = 0.48 | F_2,16_ = 5.50; **P = 0.03** | a, b, ab | F_2,16_ = 1.55; P = 0.26 |
| 4 | F_1,16_ = 7,77; **P = 0.02** | F_2,16_ = 6.39; **P = 0.02** | a, b, ab | F_2,16_ = 2.11; P = 0.18 |
| 5 | F_1,16_ = 6.60; **P = 0.03** | F_2,16_ = 5.62; **P = 0.03** | a, b, ab | F_2,16_ = 0.89; P = 0.44 |
| 6 | F_1,16_ = 6.68; **P = 0.03** | F_2,16_ = 2.09; P = 0.18 | a, a, a | F_2,16_ = 8.32; **P = 0.01** |
| 7 | F_1,16_ = 0.06; P = 0.82 | F_2,16_ = 1.89; P = 0.21 | a, a, a | F_2,16_ = 1.76; P = 0.23 |
| 8 | F_1,16_ = 17.50; **P = 0.002** | F_2,16_ = 11.66; **P = 0.003** | a, b, b | F_2,16_ = 7.08; **P = 0.01** |
| 9 | F_1,16_ = 1.09; P = 0.32 | F_2,16_ = 4.12; **P = 0.04** | a, b, ab | F_2,16_ = 3.60; P = 0.07 |
| 10 | F_1,16_ = 46.93; **P < 0.001** | F_2,16_ = 12.20; **P = 0.003** | a, b, ab | F_2,16_ = 3.81; P = 0.06 |
| **B. Canopy predators** |  |  |  |  |
| 1 | F_1,18_ = 6.63; **P = 0.02** | F_2,18_ = 0.25; P = 0.78 | a, a, a | F_2,18_ = 0.25; P = 0.78 |
| 2 | F_1,18_ = 192.23; **P < 0.001** | F_2,18_ = 0.22; P = 0.81 | a, a, a | F_2,18_ = 0.22; P = 0.81 |
| 3 | F_1,18_ = 0.94; P = 0.35 | F_2,18_ = 0.43; P = 0.66 | a, a, a | F_2,18_ = 1.75; P = 0.22 |
| 4 | F_1,18_ = 0.75; P = 0.40 | F_2,18_ = 2.21; P = 0.15 | a, a, a | F_2,18_ = 1.21; P = 0.33 |
| 5 | F_1,18_ = 0.67; P = 0.43 | F_2,18_ = 0.67; P = 0.53 | a, a, a | F_2,18_ = 2.67; P = 0.11 |
| 6 | F_1,18_ = 0.63; P = 0.44 | F_2,18_ = 0.26; P = 0.78 | a, a, a | F_2,18_ = 1.07; P = 0.38 |
| 7 | F_1,18_ = 0.19; P = 0.89 | F_2,18_ = 1.24; P = 0.32 | a, a, a | F_2,18_ = 0.22; P = 0.81 |
| 8 | F_1,18_ = 0.41; P = 0.54 | F_2,18_ = 1.37; P = 0.29 | a, a, a | F_2,18_ = 1.06; P = 0.38 |
| 9 | F_1,18_ = 0.14; P = 0.71 | F_2,18_ = 0.32; P = 0.73 | a, a, a | F_2,18_ = 0.04; P = 0.96 |
| 10 | F_1,18_ = 4.83; P = 0.05 | F_2,18_ = 2.45; P = 0.13 | a, a, a | F_2,18_ = 1.61; P = 0.24 |
| **C. Canopy pests** |  |  |  |  |
| 1 | F_1,18_ = 2.00; P = 0.18 | F_2,18_ = 0.50; P = 0.62 | a, a, a | F_2,18_ = 0.50; P = 0.62 |
| 2 | F_1,18_ = 24.65; **P < 0.001** | F_2,18_ = 5.76; **P = 0.02** | ab, a, b | F_2,18_ = 5.76; **P = 0.02** |
| 3 | F_1,18_ = 8.14; **P = 0.02** | F_2,18_ = 0.60; P = 0.56 | a, a, a | F_2,18_ = 0.01; P = 0.99 |
| 4 | F_1,18_ = 9.71; **P = 0.01** | F_2,18_ = 0.28; P = 0.76 | a, a, a | F_2,18_ = 0.83; P = 0.46 |
| 5 | F_1,18_ = 0.89; P = 0.36 | F_2,18_ = 0.73; P = 0.50 | a, a, a | F_2,18_ = 0.42; P = 0.66 |
| 6 | F_1,18_ = 2.29; P = 0.16 | F_2,18_ = 0.01; P = 0.99 | a, a, a | F_2,18_ = 0.57; P = 0.58 |
| 7 | F_1,18_ = 0.79; P = 0.39 | F_2,18_ = 0.19; P = 0.83 | a, a, a | F_2,18_ = 0.93; P = 0.42 |
| 8 | F_1,18_ = 0.03; P = 0.87 | F_2,18_ = 0.81; P = 0.47 | a, a, a | F_2,18_ = 0.15; P = 0.86 |
| 9 | F_1,18_ = 1.03; P = 0.33 | F_2,18_ = 0.05; P = 0.95 | a, a, a | F_2,18_ = 0.49; P = 0.63 |
| 10 | F_1,18_ = 1.00; P = 0.34 | F_2,18_ = 1.00; P = 0.40 | a, a, a | F_2,18_ = 1.00; P = 0.40 |

**^a^**Treatment (T, S, F): T = Tillage; S = Spontaneous cover; F = Flower-driven cover
