# Supplementary material for: Effects of Ground Cover Management on Insect Predators and Pests in a Mediterranean Vineyard
Source: Insects. 2019 Nov 23;10(12):421. doi: 10.3390/insects10120421 (PMC6956331; doi:10.3390/insects10120421)
Supplement: Supplementary file 1 [file insects-10-00421-s001.zip › Table S4.docx]

**Table S4.** Two-way ANOVA results of predator biodiversity values. Significant differences are highlighted in bold.

| **Observed diversity (^q^D)** | | **Year** | **Treatment** | **Year x Treatment** |
| --- | --- | --- | --- | --- |
| **Ground level** |  |  |  |  |
| Carabidae | ^0^D | F_1,16_ = 2.61, P = 0.14 | F_2, 16_= 4.69, **P = 0.04** | F_2,16_ = 0.47, P = 0.64 |
|  | ^1^D | F_1,16_= 0.30, P = 0.59 | F_2,16_ = 1.29, P = 0.32 | F_2,16_ = 0.44, P = 0.66 |
|  | ^2^D | F_1,16_ = 0.23, P = 0.65 | F_2,16_ = 0.82, P = 0.47 | F_2,16_ = 0.54, P = 0.60 |
| Forficulidae | ^0^D | F_1,16_ < 0.01, P = 1.00 | F_2, 16_ < 0.01, P = 1.00 | F_2, 16_ < 0.01, P = 1.00 |
|  | ^1^D | F_1,16_ < 0.01, P = 1.00 | F_2, 16_ < 0.01, P = 1.00 | F_2, 16_ < 0.01, P = 1.00 |
|  | ^2^D | F_1,16_ < 0.01, P = 1.00 | F_2, 16_ < 0.01, P = 1.00 | F_2, 16_ < 0.01, P = 1.00 |
| Staphylinidae | ^0^D | F_1,16_ = 1,08, P = 0.32 | F_2, 16_= 0.97, P = 0.41 | F_2,16_ = 0.12, P = 0.89 |
|  | ^1^D | F_1,16_= 1.66, P = 0.22 | F_2,16_ = 0.75, P = 0.49 | F_2,16_ = 0.30, P = 0.75 |
|  | ^2^D | F_1,16_ = 2.09, P = 0.17 | F_2,16_ = 0.53, P = 0.60 | F_2,16_ = 0.51, P = 0.61 |
| Potential pests | ^0^D | F_1,16_ = 0.80, P = 0.39 | F_2, 16_= 4.20, **P = 0.04** | F_2,16_ = 0.20, P = 0.82 |
|  | ^1^D | F_1,16_ = 0.80, P = 0.39 | F_2, 16_= 4.20, **P = 0.04** | F_2,16_ = 0.20, P = 0.82 |
|  | ^2^D | F_1,16_ = 0.80, P = 0.39 | F_2, 16_= 4.20, **P = 0.04** | F_2,16_ = 0.20, P = 0.82 |
| **Canopy level** |  |  |  |  |
| Aeolothripidae | ^0^D | F_1,18_ = 3.77, P = 0.08 | F_2,18_ = 0.23, P = 0.80 | F_2,18_ = 1.92, P = 0.19 |
|  | ^1^D | F_1,18_ = 4.82, **P = 0.05** | F_2,18_ = 0.14, P = 0.87 | F_2,18_ = 1.53, P = 0.26 |
|  | ^2^D | F_1,18_ = 5.30, **P = 0.04** | F_2,18_ = 0.10, P = 0.91 | F_2,18_ = 1.25, P = 0.32 |
| Chrysopidae | ^0^D | F_1,18_ = 0.25, P = 0.63 | F_2,18_ = 0.25, P = 0.78 | F_2,18_ = 1.75, P = 0.22 |
|  | ^1^D | F_1,18_ = 0.25, P = 0.63 | F_2,18_ = 0.25, P = 0.78 | F_2,18_ = 1.75, P = 0.22 |
|  | ^2^D | F_1,18_ = 0.25, P = 0.63 | F_2,18_ = 0.25, P = 0.78 | F_2,18_ = 1.75, P = 0.22 |
| Cecidomyiidae | ^0^D | F_1,18_ = 1.07, P = 0.32 | F_2,18_ = 3.27, P = 0.07 | F_2,18_ = 1.27, P = 0.32 |
|  | ^1^D | F_1,18_ = 1.13, P = 0.31 | F_2,18_ = 3.26, P = 0.07 | F_2,18_ = 1.18, P = 0.34 |
|  | ^2^D | F_1,18_ = 1.19, P = 0.30 | F_2,18_ = 3.24, P = 0.08 | F_2,18_ = 1.13, P = 0.36 |
| Coccinellidae | ^0^D | F_1,18_ = 0.11, P = 0.75 | F_2,18_ = 0.11, P = 0.90 | F_2,18_ = 1.44, P = 0.27 |
|  | ^1^D | F_1,18_ = 0.19, P = 0.67 | F_2,18_ = 0.16, P = 0.86 | F_2,18_ = 1.37, P = 0.29 |
|  | ^2^D | F1,18 = 0.25, P = 0.63 | F_2,18_ = 0.20, P = 0.83 | F_2,18_ = 1.30, P = 0.31 |
| Potential pests | ^0^D | F_1,18_ < 0.01, P = 1.00 | F_2,18_ = 0.50, P = 0.62 | F_2,18_ = 1.50, P = 0.26 |
|  | ^1^D | F_1,18_ = 0.37, P = 0.56 | F_2,18_ = 0.68, P = 0.52 | F_2,18_ = 1.32, P = 0.30 |
|  | ^2^D | F_1,18_ = 0.62, P = 0.45 | F_2,18_ = 0.81, P = 0.47 | F_2,18_ = 1.19, P = 0.34 |
